# Effects of writers, erasers and readers within miRNA‐related m6A modification in cancers

**DOI:** 10.1111/cpr.13340

**Published:** 2022-09-26

**Authors:** Huiru Feng, Xiaofei Yuan, Shuting Wu, Yue Yuan, Linchong Cui, Danfan Lin, Xiaohong Peng, Xiong Liu, Fan Wang

**Affiliations:** ^1^ Department of Otolaryngology‐Head and Neck Surgery, Nanfang Hospital Southern Medical University Baiyun District Guangzhou People's Republic of China

## Abstract

**Background:**

As one of the most abundant post‐transcriptional mRNA modifications, N6‐methyladenosine (m6A) has attracted extensive attention from scientists. Emerging evidence indicates that m6A modification plays a significant role in cancer‐related signalling pathways. Existing research demonstrates that m6A modifications were also identified in miRNAs and contribute to cancer‐related signalling pathways.

**Methods:**

A literature retrieval has been performed to collect m6A‐miRNA‐related original articles published in recent years. Later, a systematic analysis has been conducted to abstract and classify the relationships between m6A modification and miRNAs, and their contributions to tumorigenesis and cancer development.

**Results:**

Accumulating literature provides important insights into multiple relationships between m6A modifications and miRNAs. Mechanically, m6A writer and eraser alter pri‐miRNAs m6A levels, and m6A readers could dually modulate pri‐miRNAs processing and pri‐miRNAs degradation. It is also been demonstrated that miRNAs impair m6A regulators' translation to influence m6A medication function in return. Aberrant expressions of m6A regulators and miRNAs could dysregulate proliferative, apoptosis, cell adhesion‐related, and malignant transformation signalling pathways, and contribute to tumour occurrence and development.

**Conclusion:**

This review summarizes the interrelationship between m6A modification and miRNAs; highlights the combined effects of each type of m6A regulator and miRNAs in cancers. These findings enhance our understanding of m6A‐miRNAs' multiple interactions and significant modulatory role in tumorigenesis and progression.

## Graphical abstract

The interactions between m6A modification and miRNAs mainly consist of the following patterns, m6A modification mediates pri‐miRNAs processing (A), miRNAs target 3′s UTR of m6A regulators (B), miRNAs orchestrate with m6A regulators to exert their intrinsic functions (C). Since interactions between m6A modification and miRNAs contribute to cancer‐related pathways, dysregulations of these interactions cause a series of alterations in diverse cancers.

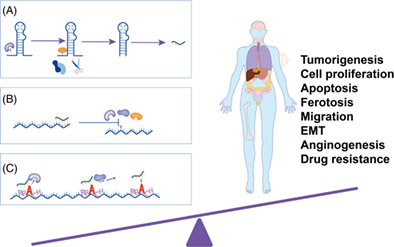

## 1 INTRODUCTION

**FIGURE 1:**
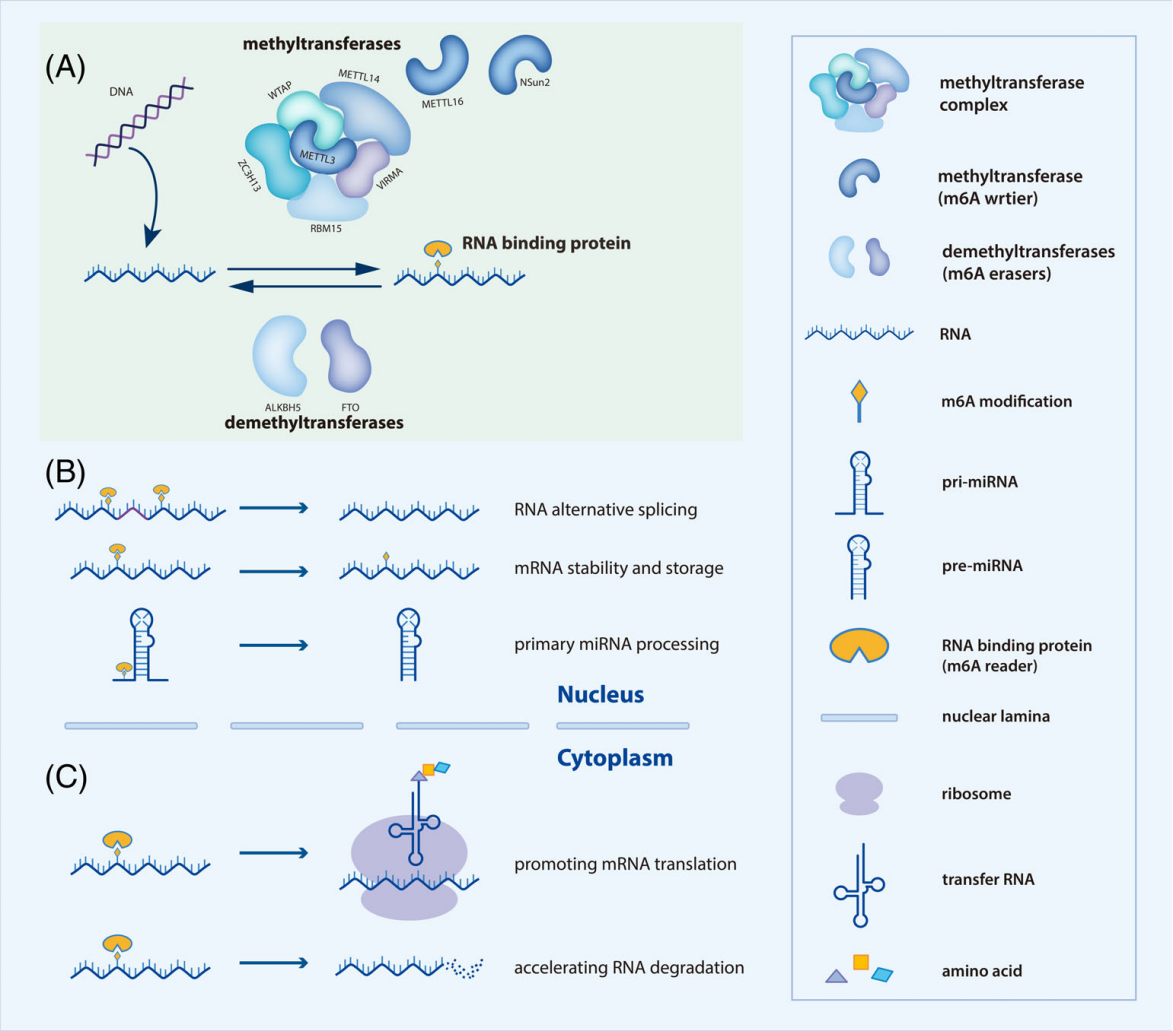
The dynamic process of m6A modification and diverse functions of m6A readers. (A) Methyl was installed at N6 of adenosine by m6A methyltransferase complex which consists of METTL3, METTL14, WTAP, RBM15, VIRMA and ZC3H13. In addition, METTL16 and NSun2 could function individually. M6A modification could be removed by demethyltransferases FTO and ALKBH5. m6A writers and erasers dynamically regulate RNA m6A levels, followed by recognition of m6A binding proteins. Different readers manipulate diverse RNA metabolism processes. (B) In the nucleus, readers could mediate mRNA alternative splicing, maintain mRNA stability and primary miRNAs processing. (C) In the cytoplasm, readers could promote mRNAs translation and the exact opposite function, mRNAs degradation

N6‐methyladenosine (m6A) modification is the most prevalent post‐transcriptional modification in mammal's mRNAs. It refers to that methylation occurs in the N6‐position of adenosine.^1^, ^2^ As the development of high throughput sequencing technology accelerates, m6A is found to be installed at adenosine within RRACH (R corresponds to G or A; H corresponds to A, C or U) motif, which is commonly located at the 3′ untranslated region (UTR), intron and stop codon of the mRNAs.^3^, ^4^ Mechanically, m6A modification is a reversible process, dynamically regulated by methyltransferases, demethyltransferases and RNA binding proteins, namely writers, erasers and readers, respectively.^5^ Writers and erasers are responsible for the installation and dismantlement of methyl, counterbalancing the levels of m6A modification (Figure 1A). As for readers, they could recognize the m6A modification and manipulate the directions of RNA metabolism, including translation, stabilization, degradation, alternative splicing, pri‐miRNAs processing and so on (Figure 1B,C). Functionally, m6A modification directly mediates RNA fate to influence proteins, main executors in the life processes, and hence drives a series of subsequent effects on cell biological functions.^6^, ^7^ Extensive studies have elucidated that m6A regulators contribute to variant cell signalling pathways, having profound impacts on disease, stem cell differentiation, cell proliferation, tumorigenesis, cancer metastasis, invasion and clinical prognosis.^8^, ^9^, ^10^

miRNAs, one of the non‐coding RNAs, are a type of bioactive molecules consisting of approximately 22nts single‐strand nucleotides. Functionally, miRNAs drive an interfering effect on mRNAs translation and induce mRNAs degradation by targeting mRNA 3′UTR via the complementary base pairing principle.^11^, ^12^, ^13^ Previous studies have well‐validated that miRNAs reflect disturbing effects on cancer‐associated mRNAs, exhibiting influence covering cancer stem cell differentiation, proliferation, metastasis, prognosis and therapeutic value in various cancers in the past decades.^14^, ^15^, ^16^, ^17^

In addition to mRNAs, miRNAs are proven to be widespread modified with N6‐methyl, which are commonly enriched in the consensus sequence of GGAC.^18^ In 2015, Alarcon C. R and colleagues corroborated that primary miRNA (pri‐miRNA) processing was associated with an m6A‐dependent manner, raising the curtain on the m6A regulatory mechanism of miRNAs processing and its effect in various cancers.^18^ On the other hand, numerous studies have found that miRNAs could alter m6A levels in turn by suppressing the expression of m6A regulators. Emerging investigations have shed light on the molecular basis and effects of m6A and miRNAs interactions. Nevertheless, detailed mechanisms regarding the roles of each type of m6A regulator that contribute to tumour pathological have not yet been completely realized. In this case, it is necessary to comprehensively understand the relationship between m6A and miRNAs from available reports, laying a systematic and theoretical foundation to analyse joint effects on cancer occurrence and development.

In this review, we aim to introduce the basic knowledge of m6A modification, sum up the interaction between m6A regulators and miRNAs, emphasize the contributions of each regulator to cancer physical functions when cooperating with miRNAs and expect to inform future clinical applications.

## 2 BIOLOGICAL FUNCTIONS OF M6A REGULATORS

M6A modification is modulated by three types of m6A regulators consisting of methyltransferases, demethyltransferases and RNA‐binding proteins three types of proteins, namely m6A writers, erasers and readers (Figure 1A, Table 1).

**TABLE 1:** Functions of m6A regulators

Type	Regulator	Function	Ref.
Writer	METTL3	Catalyses m6A modification	19
	METTL14	Provides a platform for METTL3's catalysis	20
	METTL16	Catalyses m6A modification	27
	WTAP	Binds to METTL3‐METTL14 complex and recruits it to nuclear speckle	21, 22
	VIRMA	Recruits METTL3‐METTL14‐WTAP complex to 3′UTR and stop codon region	23
	RMB15/RMB15B	Connect with the METTL3‐WTAP complex for XIST silencing	24, 25
	ZC3H13	Bridge RBM15 to WTAP‐VIRMA complex	26
	NSun2	Catalyses m6A modification	28
Eraser	FTO	Removes m6A modification	29, 30
	ALKBH5	Removes m6A modification	31
Reader	YTHDF1	Accelerates translation or promotes stability and storage of transcripts	33
	YTHDF2	Promotes mRNAs and pre‐miRNAs decay	34
	YTHDF3	Coordinates with YTHDF1 and YTHDF2 to exert their own effects	35
	YTHDC1	Assists mRNA precursor export to the cytoplasm	36, 37
	YTHDC2	Enhances translation efficiency and decreases mRNA abundance; decreases target mRNAs translation	38, 39
	HNRNPA2B1	Mediates mRNAs alternative splicing and pri‐miRNAs processing	41
	HNRNPC	Mediates mRNAs splicing	33
	IGF2BP1	Promotes mRNAs translation and stability	42
	EIF3	Promotes mRNAs translation	43
	NKAP	Mediates pri‐miRNAs processing	44

### 2.1 M6A writers

M6A modification is installed by methyltransferase multicomponent involving a catalytic subunit methyltransferase‐like enzyme 3 (METTL3),^19^ a stabilize subunit methyltransferase‐like enzyme 14 (METTL14),^20^ Wilm's tumour‐associated protein (WTAP), vir‐like m6A methyltransferase‐associated (VIRMA, also known as KIAA1429), RNA‐binding motif protein 15 (RBM15) and its homologue (RMB15B), zinc finger CCCH‐type containing 13 (ZC3H13) and so on (Figure 1A). Somehow, some methyltransferases function by themselves, for instance, METTL16 and NSun2. METTL3, METTL14 and METTL16 all belong to the methyltransferase‐like family. METTL14 forms tight conjunction with catalytic METTL3 as a stable heterodimer, providing a platform for METTL3's catalysis. The rest of the components depend on the METTL3‐METTL14 core complex, devoting to the activity and localization of the writer complex. WTAP interacts with the METTL3‐METTL14 complex and guides it to nuclear speckle.^21^, ^22^ VIRMA recruits METTL3‐METTL14‐WTAP to 3′ UTR and regions near the stop codon for m6A location‐specific installation.^23^ RMB15 and its paralog RMB15B are revealed to connect with the METTL3‐WTAP complex, which is necessary for m6A‐dependent X‐inactive‐specific transcript (XIST) silencing to mediate X chromosome inactivation.^24^, ^25^ ZC3H13 is required for adenosine methylation by bridging RBM15 to the WTAP‐VIRMA complex.^26^ METTL16 is a novel m6A methyltransferase that modifies the U6 snRNA, other pre‐mRNAs and various non‐coding RNAs independently.^27^ NSun2 is one of the tRNA methyltransferases, it is also found to exhibit RNA m6A methylation function in colon‐rectal cancer.^28^ Given all that has been mentioned so far, it is demonstrated that m6A methylation is manipulated by complicated machinery, which is orchestrated by various methyltransferases for adenosine methyl installation.

### 2.2 M6A erasers

For now, demethyltransferases only account for a few parts of m6A regulators. FTO and ALKBH5, two acknowledged and well‐researched demethyltransferases, discharge methyl from adenosine (Figure 1A). FTO, fat mass and obesity‐associated protein, was firstly reported to be associated with fatty acid biogenesis and the first demethyltransferase.^29^ Especially, FTO was mentioned to mediate small RNA demethylation, for example, miRNAs. In addition to m6A demethylation, FTO was shown to modulate m6Am and m1A demethylation.^30^ FTO belongs to the AlkB family, thus ALKBH5 was identified by Zheng et al who decided to figure out whether other AlkB homologues are acting as demethyltransferase other than FTO.^31^ Methyltransferase and demethyltransferase counterbalance the m6A modification level in a highly dynamic manner, potentially contributing to disease and cancers associated imbalance.

### 2.3 M6A readers

M6A readers are a type of RNA‐binding protein that can decipher the m6A mark and specifically manipulate RNA metabolism, dominating the downstream biological functions. In general, there are mainly two types of m6A readers, YT521‐B homology (YTH) domain and heterogeneous nuclear ribonucleoproteins (HNRNPs). YTH domain consists of YTH domain family 1–3 (YTHDF1‐3) and YTH domain containing 1–2 (YTHDC1‐2) in humans.^32^ In terms of YTHDF1‐3, they could dominate transcripts into exactly opposite destinies. YTHDF1 accelerates translation or promotes stability and storage of transcripts.^33^ On the contrary, YTHDF2 expedites mRNA decay and impairs its enrichment.^34^ As for YTHDF3, it coordinates with YTHDF1 or YTHDF2 to exert their effects on corresponding m6A‐containing mRNAs.^35^ YTHDC1 assists mRNA precursor export to the cytoplasm for further maturation and translation.^36^, ^37^ YTHDC2 exerts a critical effect in mammalian spermatogenesis by enhancing translation efficiency and decreasing mRNA abundance.^38^ In contrast to the former finding, YTHDC2 is found to facilitate mRNA degradation and hence decrease translation efficiency.^39^

Another group of HNRNPs, including HNRNPC, HNRNPG and HNRNPA2B1, is characterized by alternative splicing, transcript processing, local RNA structure remodelling and RNA‐protein interaction modulation.^40^ Among these HNRNPs, HNRNPA2B1 is found to directly bind to m6A and mediate primary miRNA processing with miRNA microprocessor complex.^41^ Apart from these two main family m6A readers, insulin‐like growth factor‐2 mRNA‐binding proteins (IGF2BPs) are found to be a new group of m6A readers. Compelling evidence revealed that IGF2BPs facilitate mRNAs translation and stability.^42^ Some known molecules are also identified as m6A readers. Eukaryotic initiation factor 3 (eIF3) is elucidated to initiate translation through recruiting 43S ribosomal complex via binding m6A containing 5′UTR mRNAs in a cap‐independent manner.^43^ Nuclear factor κB ‐related protein (NKAP) could exhibit miRNAs biogenesis assistance effect in a similar pattern of HNRNPA2B1.^44^ Beyond all that, m6A readers' functions are much more than available evidence, deeper investigation is recommended for general understanding.

## 3 INTERACTION PATTERNS BETWEEN M6A MODIFICATION AND MIRNAS

**FIGURE 2:**
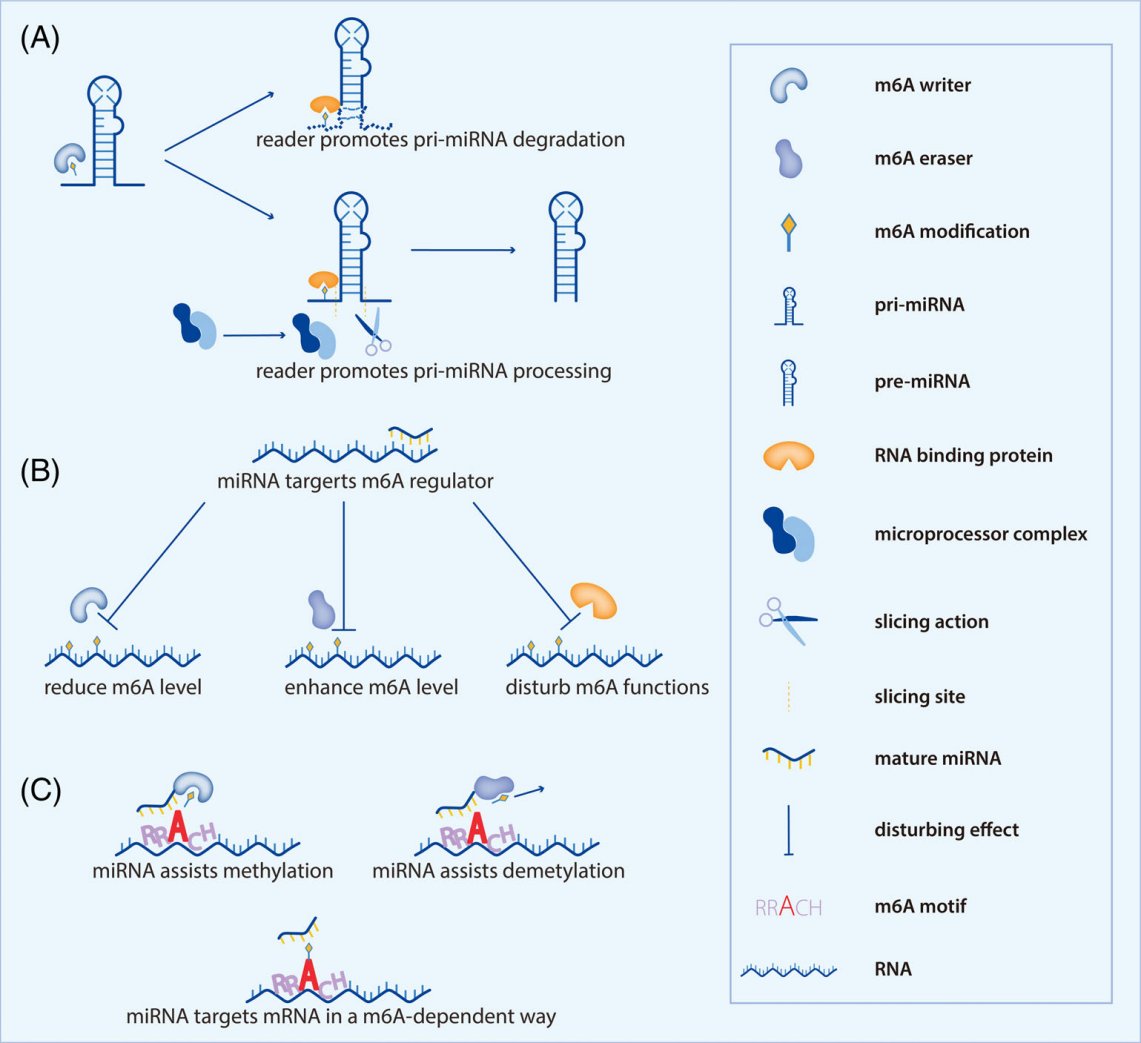
The patterns of interaction between m6A modification and miRNAs. (A) m6A modification mediates pri‐miRNAs processing. m6A writer deposits methyl at the m6A motif of pri‐miRNA followed by m6A reader recognizing it. Part of readers like HNRNP2B1, NKAP and YTHDC1 recruit microprocessor complex for flanking slicing, subsequently receiving a precursor miRNA, while YTHDF2 could accelerate pre‐miRNA degradation. (B) miRNAs target 3′s UTR of m6A regulators. miRNAs could induce m6A level alteration or impede m6A reader function implementation through targeting m6A regulators' 3′ UTR. (C) miRNAs orchestrate with m6A regulators to exert their intrinsic functions. miRNAs could assist m6A methylation and demethylation by interacting with the writer and eraser respectively. miRNA targets mRNA 3′UTR in an m6A‐dependent way

From the available literature, we found multiple interactions between m6A modification and miRNAs. We develop the following interaction patterns for better understanding (Figure 2).

### 3.1 M6A modification mediates pri‐miRNAs processing (Figure 2A)

miRNA maturation undergoes a series of stages. Primary miRNAs, transcribed from the genome, include 100s to 1000s of nucleotides and basically consist of at least one loop‐stem‐flanking region. Primary miRNAs require flanking slicing to form precursor miRNAs, which were manipulated by microprocessor complex DGCR8 and DROSHA. Next, precursor miRNAs that contain the stem structure of the mature part of miRNA/miRNA* duplex at the opposite side and the loop structure are exported outward cytoplasm by exportin5‐RAN‐GTP complex. In the cytoplasm, DICER removes the loop structure of precursor miRNAs, followed by AGO cleaving the leaving miRNA/miRNA* duplex into single‐stranded mature miRNAs.^45^, ^46^ In 2015, Alarcon et al firstly investigated the role of METTL3 toward primary miRNA processing.^18^ It is confirmed that METTL3 enhanced pri‐miRNAs m6A abundance and miRNAs expressions. Researchers proposed that m6A modification initiated the interaction between DGCR8 and m6A‐containing pri‐miRNAs for slicing during the processing. On the contrary, demethyltransferase ALKBH5 has been reported to suppress pri‐miR‐193a processing.^47^ Recently, Zhang and colleagues revealed that overexpressed‐METTL3 elevated the pri‐miR‐25 m6A level, and subsequently reader NKAP bound with m6A and recruited DGCR8 for further processing.^44^ These findings illustrated a pri‐miRNA processing pattern that is featured by the m6A modification and reader‐dependent foundation for microprocessor complex recruitment, providing a more complete theory frame for understanding the effect of m6A modification on miRNAs synthesis modulation.

### 3.2 miRNAs target 3′s UTR of m6A regulators (Figure 2B)

It is well‐validated that miRNAs as vital bioactive molecules, aim at disturbing the translation of targeted mRNAs. In this way, miRNAs could base pair with N6‐methyladenosine regulators' mRNAs followed by downstream m6A modification alteration. MiR‐483p has been found to target METTL3 3′UTR to reduce the m6A level of p21, driving an antiproliferation effect in breast cancer.^48^ Similarly, miR‐193a‐3p could repress ALKBH5 expression, thus elevating AKT2 m6A abundance and inhibiting apoptosis in glioma.^49^ Though miRNA could not change m6A amounts when repressing readers' expression, it does influence readers' function implementation. It is uncovered that MALAT1‐sponged‐miR‐204 failed to bind to reader IGF2BP2. Hence, elevated‐IGF2BP2 could recognize m6A‐containing MYC and promote translation, exhibiting an oncogenic effect in thyroid cancer.^50^ Therefore, the disturbing effect of miRNAs could exhibit global impacts on cell m6A modification as well as downstream biological functions, providing an overall insight into the bidirectional relationship between m6A modification and miRNAs.

### 3.3 miRNAs orchestrate with m6A regulators to exert their intrinsic functions (Figure 2C)

Recently, miRNAs are somehow identified as possible readers of m6A motifs, assisting writers and erasers to better exert functions of methylation and demethylation respectively. miRNAs binding sites are discovered to be enriched at m6A motifs of mRNAs 3′UTRs, which is a fundamental basis that miRNAs mediate m6A regulators to be located at the m6A motifs. A report carried out by Chen et al suggested that miRNAs orchestrated METTL3's recognition of the m6A motif for methyl installation. Further examination confirmed that overexpression of miRNAs mediated METTL3 to bind with mRNAs m6A motifs for methylation.^51^ And not coincidentally, John P. Zepecki and colleagues revealed that miR‐145 elevated the demethylation activity of FTO by forming FTO/AGO1/ILF3/miR‐145 complex. Further investigation demonstrated that miR‐145 induced FTO's binding to mRNAs and reduced the m6A level of target mRNAs during glioma stem cells (GSCs) state transition to differential glioma cells (DGCs).^52^ For both reports of miRNAs assisting catalysis of METTL3 or FTO, overexpression or downregulation of miRNAs do not cause expression alteration of these m6A writers and erasers. It is miRNAs that modulate localization and binding of METTL3 and FTO to more perfectly perform their functions. Another novel study conducted by Junmei Cheng et al indicated that m6A modification in MYCN is indispensable for miR‐98's binding to MYCN 3′UTR. Mutations of the m6A motif of MYCY impeded the interaction between miR‐98 and MYCN. Hence, over‐expressed pseudo reader miR‐98 disturbed MYCN expression in a novel m6A‐dependent way.^53^ These findings supported the phenomenon that miRNAs could be viewed as a kind of feasible readers of m6A motifs, showing more possibility of regulative patterns between miRNAs and m6A modification.

## 4 COMBINED ROLES OF M6A WRITERS AND MIRNAS IN CANCERS

### 4.1 Methyltransferases regulate pri‐miRNAs processing

After Alarcon's research concerning METTL3 and m6A set a research foundation and reference for m6A‐dependent miRNAs processing, accumulating studies have been conducted and showed that writer‐mediated miRNAs maturation exhibited generality of aggressive phenotypes in various cancers. Next, we will illustrate that m6A writers' functions via mediating miRNAs expression from two diverse biological aspects, cancer proliferation and metastasis (Table 2).

**TABLE 2:** M6A writers regulate miRNAs processing

M6A regulators	Cancer type	miRNA	Writers' role	Mechanism	Validation approach/model	Function of writers	Ref
METTL3	Gallbladder cancer	miR‐92b‐3p	Oncogene	↑	MeRIP‐qPCR, in vitro pri‐miRNA processing assays (cell)	Activate PTEN/PI3K/AKT pathway	54
METTL3	Ovarian cancer	miR‐126‐5p	Oncogene	↑	MeRIP‐qPCR (cell)	Activate PTEN/PI3K/AKT/mTOR pathway	55
METTL3	Bladder cancer	miR‐221/222	Oncogene	↑	MeRIP‐qPCR (cell)	Inhibit PTEN expression	56
METTL3	Cervical cancer	miR‐193b	Oncogene	↑	MeRIP‐qPCR (cell)	Stimulate CCND1 expression	57
METTL14	CRC	miR‐375	Suppress	↑	MeRIP‐qPCR, in vitro pri‐miRNA processing assay (cell)	Stimulate YAP1 and SP1 expression	58
METTL3	PDAC	miR‐25‐3p	Oncogene	↑	MeRIP‐qPCR, in vitro pri‐miRNA processing assays (cell)	Activate PHLPP2/AKT‐p70S6K pathway	44
METTL3	Lung cancer	miR‐143‐3p	Oncogene	↑	MeRIP‐qPCR (cell)	Inhibit VASH1 expression and promote angiogenesis and tublin depolymerization	59
METTL14	HCC	miR‐126‐5p	Suppress	↑	MeRIP‐qPCR (cell)	Suppresses tumour invasion and metastasis	85
METTL14	Breast cancer	miR‐146a‐5p	Oncogene	↑	MeRIP‐qPCR (cell)	Promote EMT	61
METTL3	CRC	miR‐1246	Oncogene	↑	MeRIP‐qPCR(cell)	Inhibit SPERD2 expression and stimulate MAPK pathway	60
METTL3	Gastric cancer	miR‐17‐92 cluster	Oncogene	↑	MeRIP‐qPCR (cell)	Inhibit PTEN/TMEM127 and stimulate AKT/mTOR pathway	81
METTL3	Breast cancer	miR‐221‐3p	Oncogene	↑	MeRIP‐qPCR (cell)	Stimulate drug resistance‐related HIPK2/Che1	82
NSun2	CRC	miR‐125b	Oncogene	↓	In vitro pri‐miRNA processing assay (cell)	Inhibit Gab2 expression and stimulate PI3K/AKT pathway	28

Abbreviations: CRC, colorectal cancer; EMT, epithelial‐mesenchymal transition; HCC, hepatocellular cancer; MeRIP‐qPCR, methylated RNA immunoprecipitation quantitative polymerase chain reaction; NSCLC, non‐small cell lung cancer; ↑, promote pri‐miRNA processing; ↓, inhibit pri‐miRNA processing.

The limitless replicative potential is one of the most prominent features of tumours. In gallbladder cancer, it is elucidated that reduced‐deoxycholic acid (DAC) failed to disrupt METTL3 assemble with methyltransferase complex, assisting METTL3‐m6A‐dependent pri‐miR‐92b processing. In this situation, miR‐92b‐3p silenced tumour suppressor PTEN and subsequently stimulated PI3K/AKT pathway, fostering cell growth and exerting an oncogenic effect in gallbladder cancer.^54^ In pancreatic duct adenocarcinoma (PDAC), cigarette smoke condensate (CSC)‐induced METTL3 promoted proliferation, driving an oncogenic function. METTL3 accelerated m6A‐containing pri‐miR‐25 processing by recruiting a microprocessor DGCR8 with recognition of m6A reader NKAP. Consequently, miR‐25‐3p inhibited PHLPP2 expression followed by an evocation of AKT‐p70S6K signalling.^44^ In ovarian cancer, elevated METTL3 was found to contribute to miR‐126‐5p maturation and exert a tumour‐promoting effect. Excessive miR‐126‐5p stimulated PTEN/PI3K/Akt/mTOR pathway, promoting cancer cell proliferation as well as suppressing apoptosis.^55^ In bladder cancer, upregulated METTL3 was involved in facilitating pri‐miR‐221/222 processing to encourage cell proliferation. Enhanced mature miR‐221/222 directly bound to PTEN mRNA, exerting an oncogenic effect.^56^ So far, these observations confirmed that the well‐researched molecule METTL3, seemed to have a close connection with PTEN/PI3K/AKT/mTOR pathway. METTL3 utilized different miRNAs as a bridge to drive cell proliferation and tumour‐promoting effect in an m6A‐based pattern. Furthermore, it is indicated that METTL3 has a prevalent modification function toward diverse pri‐miRNAs, suggesting the ubiquitous role of m6A modification in miRNA metabolism.

Apart from cell proliferation‐related signalling, m6A writers could mediate cell cycle protein and proliferation‐associated protein to facilitate cancer cell growth via utilizing m6A‐related miRNAs synthesis. In cervical cancer, it is elucidated that METTL3 could promote pri‐miR‐193b m6A level and processing. However, METTL3 was reduced in cervical cancer, hence failed to elevate mature miR‐193b and undermine the silencing effect of miR‐193b on cell cycle protein CCND1.^57^ In contrast to the findings described above, METTL3 exerted an infrequent tumour‐suppressing effect in this article by negatively regulating CCND1. Though only METTL3 exhibits catalysis, METTL14 was validated to be crucial for methylation. METTL14 was also confirmed to be reduced and associated with a tumour‐suppressing effect in colorectal cancer (CRC). METTL14 was found to be responsible for facilitating miR‐375 processing. Since miR‐375 targeted YAP1, a proliferation‐associated protein, METTL14 eventually repressed cancer cell proliferation in CRC.^58^

Tissue invasion and metastasis are other predominant features of aggressive cancer. It has been widely uncovered that m6A writer‐modulated miRNAs could regulate EMT (epithelial‐to‐mesenchymal transition), angiogenesis, metastasis‐associated molecules, and signal transduction pathways, contributing to tumour distant metastasis and undesirable prognosis. In lung cancer, overexpressed‐METTL3 was involved in vasohibin‐1(VASH1)‐induced brain metastasis by facilitating pri‐miR‐143 processing. Functionally, VASH1 mediated angiogenesis and tubulin depolymerization through regulating vascular endothelial growth factor‐A (VEGFA) degradation and tubulin detyrosination. So METTL3/miR‐143 aggravated hematogenous metastasis and migration to offer a proper opportunity for brain metastasis.^59^ SPRED2 was elucidated to function as a tumour‐suppress regulator and repress metastasis‐relevant MAPK pathway in various cancers. In CRC, it is reported that METTL3 modulated miR‐1246 maturation and promoted aggressive phenotype. miR‐1246 suppressed SPRED2 expression, exerting metastasis‐facilitating function via MAPK pathway.^60^ In breast cancer, aberrant elevated METTL14 promoted migration and invasion through EMT via reshaping the miRNAs profile. Lately, hsa‐miR‐146a‐5p was identified as a downstream regulative objective of METTL14 and was enriched in the cell adhesion aspect through bioinformatics analysis.^61^

The evidence presented in this section depicted a general phenomenon that writers increase pri‐miRNAs m6A levels followed by facilitating pri‐miRNAs processing. Writers are capable of mediating tumorigenesis, cancer cell growth and distant metastasis by enhancing miRNA levels. However, in proteinase‐activated receptor 2 (PAR2)‐correlated CRC, one unanticipated finding was that another writer NSun2 inhibited miR‐125 processing while elevating the pri‐miR‐125 m6A level. Eventually, NSun2 intensified PAR2‐correlated CRC invasion capacity via activating the miR‐125/Gab2/PI3K/AKT axis, exerting an oncogenic effect.^28^ Unlike the prevalent writer characteristic of facilitating miRNAs processing, this article is worth thinking over the radical roles of m6A writers in pri‐miRNAs processing. It is required more evidence to support writers' mutual modulations of miRNAs maturation. These not only indicated the close connection between m6A writers and cancer aggressive phenotypes but also provided more comprehensive insights into the way that we view writers' effect on pri‐miRNAs processing.

### 4.2 miRNAs target methyltransferases

Plenty of attempts have been made to demonstrate that miRNAs participate in reversing m6A modification amount, which is theoretically able to reflect on downstream pathways and biological functions via directly binding with m6A regulators' mRNAs in various cancers (Table 3).

**TABLE 3:** miRNAs target m6A writers 3′UTR

miRNA	Cancer type	M6A regulators	Writers' role	Validation approach/model	Function of writers	Ref
miR‐33a	NSCLC	METTL3	Oncogene	Dual luciferase report assay (cell)	Stimulate EGFR pathway	62
miR‐338‐5p	NSCLC	METTL3	Oncogene	Dual luciferase report assay (cell)	Enhance c‐MYC m6A level and expression	63
miR‐186	Hepatoblastoma	METTL3	Oncogene	Dual luciferase report assay, miRNA mimic/inhibitor (cell, mouse)	Stimulate Wnt/β‐catenin pathway	64
miR‐4429	Gastric cancer	METTL3	Oncogene	Dual luciferase report assay, miRNA mimic/inhibitor (cell)	Increase SEC62 expression and disturb ER apoptosis pathway	65
let‐7g	Breast cancer	METTL3	Oncogene	Dual luciferase report assay, miRNA mimic/inhibitor (cell)	Promote proliferation and inhibit apoptosis	86
miR‐600	LUAC	METTL3	Oncogene	Dual luciferase report assay, miRNA mimic/inhibitor (cell)	Stimulate PI3K/AKT/Bcl2 to disturb mitochondrial apoptosis	66
miR‐4443	NSCLC	METTL3	Suppress	Dual luciferase report assay, miRNA mimic/inhibitor (cell)	Inhibit ferroptosis‐related FSP1 to suppress ferroptosis and impair cisplatin efficacy	67
miR‐193b‐5p	Gastric cancer	METTL3	Oncogene	Dual luciferase report assay, miRNA mimic/inhibitor (cell)	Promote gastric cancer progression	87
miR‐186	Oesophageal cancer	METTL3	Oncogene	Dual luciferase report assay, miRNA mimic/inhibitor (cell)	Alter m6A level globally, promote proliferation and inhibit apoptosis	88
miR‐139‐5p	HCC	WTAP	Oncogene	Dual luciferase report assay, miRNA mimic/inhibitor (cell)	Promote EMT	68

Abbreviations: EMT, epithelial‐mesenchymal transition; HCC, hepatocellular cancer; LUAC, lung adenocarcinoma; NSCLC, non‐small cell lung cancer.

Current studies have uncovered that as reduced‐miRNAs failed to target METTL3 in diverse cancers, augmented METTL3 elevated proliferative‐correlative proteins and signalling pathways in a miRNAs‐METTL3‐related way. In NSCLC, miR‐33a was validated to be decreased and attenuated aggressive tumour phenotype via disturbing METTL3 expression. Further exploration revealed that METTL3 stimulated the epithelial growth factor receptor (EGFR) pathway, a type of self‐sufficiency growth signalling. Hence reduced miR‐33a substantially restored METTL3 oncogenic effect through retrieving EGFR pathway.^62^ Similarly, it is also revealed that METTL3 referred to the miR‐338‐5p candidate target in NSCLC. Dramatically decreased miR‐338 rescued METTL3 expression. Differently for this time, METTL3 enhanced a proliferative‐associated protein c‐MYC expression, facilitating proliferation and migration function in lung cancer cells.^63^ In hepatoblastoma, bioinformatic software discovered miR‐186 as a regulatory molecule of METTL3. METTL3 is revealed to be particularly capable of triggering the multiplication‐associated Wnt/β‐catenin pathway. miR‐186 and METTL3 jointly contributed to cell proliferation as well as distant migration in hepatoblastoma.^64^ In breast cancer, metformin (a traditional diabetes drug) induced miR‐483‐3p expression and miR‐483‐3p was validated to target METTL3. Reduced‐METTL3 downregulated m6A‐p21level but promoted p21 expression, a cycle inhibition‐associated molecule. Therefore, metformin/miR‐483‐3p alleviated METTL3's oncogenic effect, promoting anti‐proliferation activity in breast cancer.^48^ As the articles mentioned above, various miRNAs could be available to bind to METTL3 3′UTR. A possible explanation for this might be that multiple miRNAs share similar sequences with METTL3 3′UTR, building a mutual and complicated communication in the m6A‐miRNAs research field.

Similar to cell interminate growth, resisting cell death is another distinct way leading to tumorigenesis and growth. The miRNAs‐METTL3 regulatory mechanism also participates in cancer cell death control. Cell apoptosis is the most prominent death form, mainly consisting of endoplasmic reticulum (ER), mitochondrial and death receptor pathways. SEC62 is a negative key transport molecule within ER apoptosis pathway. In gastric cancer, decreased‐miR‐4429 weakened silencing effect of oncogenic METTL3. Elevated‐METTL3 enhanced m6A‐SEC62 followed by the recognition of translation‐promoting reader IGF2BP1. Reduced miR‐4429 accelerated SEC62 expression and suppressed ER apoptosis via the METTL3/m6A/IGF2BP1 axis.^65^ And anti‐apoptosis Bcl2 is a crucial initiator of mitochondrial apoptosis. In lung adenocarcinoma (LUAD), miR‐600 could suppress METTL3 expression, and simultaneously METTL3 was capable of stimulating PI3K/AKT/Bcl2 pathway. Therefore, repressed‐miR‐600 successfully restored METTL3 level, eventually alleviating mitochondrial apoptosis and leading to LUAD progression.^66^

Compared with apoptosis, ferroptosis is a pathological, fer‐dependent and more intense necrosis death. Since apoptosis deficiency in many cancers, ferroptosis might be a potential tumour therapeutic target. It is illustrated that miR‐4443 was enriched in exosomes of cisplatin‐resistant NSCLC and was also found to target METTL3 3′UTR. Decreased‐METTL3 repressed m6A‐dependent ferroptosis suppressor protein 1(FSP1) expression. Hence, miR‐4443 suppressed NSCLC cancer ferroptosis and impaired cisplatin efficacy in a METTL3‐FSP1‐silencing way.^67^ Contrary to the former literature, it is the first time to be observed that METTL3 functioned as a tumour suppressor in a miRNA‐targeted way in this study.

Though WTAP does not possess a catalytic effect as well, it is critical to preserve catalytic function. WTAP was also implied to drive a significant role in cancer progression. In HCC, miR‐139 functioned as a negative regulator of WTAP, which was revealed to be associated with stimulation of the EMT pathway. Later, it is further confirmed that alleviated miR‐139 restored WTAP expression, facilitating HCC EMT proceeding.^68^ In accordance with the available combination of findings concerning METTL14 and WTAP, the significance of the integrality of the m6A methyltransferase complex is further supported.

From cancer cell proliferation, cell death, invasion and metastasis, writers and various miRNAs infiltrated into cancer diverse aspects, driving significant impacts on tumour characteristics. Notably, research about the roles of methyltransferase in cancers is like a dual‐edge sword, presenting both oncogenic and tumour‐suppressing effects with different writer‐miRNA combinations. Based on available limited data, though METTL3 has a preference to exert an oncogenic effect, it is worthwhile mentioning that METTL14 exhibits dual effects but tends to present a protective, tumour‐suppressing role in cancers. However, until now scientists are incapable of providing a reasonable explanation. Understandably, they did not possess decisive effects since m6A modification and miRNAs both function as post‐transcriptional modifications. Even so, scientists are still looking forward to discovering some regularity or inclination for future applications.

## 5 COMBINED ROLES OF M6A ERASERS AND MIRNAS IN CANCERS

Demethyltransferases are responsible for removing N6‐methyl from pri‐miRNA, reversing the effect of methyltransferase. However, demethyltransferases do not always drive the opposite function against methyltransferase's common pri‐miRNAs processing‐promoting effect. So far, the discovery of demethyltransferase is at a standstill, consistent with the low production of relative research articles. Literature regarding FTO and AKLBH5 constitutes the whole available evidence but is virtually the tip of the iceberg (Table 4).

**TABLE 4:** The interaction between m6A erasers and miRNAs

M6A regulators	Cancer type	miRNA	Erasers' role	Mechanism	Validation approach/model	Function of erasers	Ref
FTO	Breast cancer	miR‐181‐3p	Oncogene	Processing↓	Not in m6A‐dependent way	Promote metastasis‐related adhesion	69
FTO	NSCLC	miR‐607	Oncogene	miRNA target erasers 3′UTR	Dual luciferase report assay, miRNA mimic/inhibitor (cell)	Promote tumorigenesis and invasion	70
ALKBH5	Osteosarcoma	miR‐181b‐3p	Suppress	Processing↑	MeRIP‐qPCR (cell)	Inhibit YAP expression	71
ALKBH5	Oesophageal cancer	miR‐193a‐3p	Oncogene	Processing↑ miRNA target erasers 3′UTR	MeRIP‐qPCR, dual luciferase report assay (cell)	Promote cancer cell proliferation and metastasis	47
ALKBH5	Glioma	miR‐193a‐3p	Suppress	miRNA target erasers 3′UTR	Dual luciferase report assay, miRNA mimic/inhibitor (cell)	Stimulate AKT2/Bcl2/survivin, inhibit intrinsic apoptosis	49
ALKBH5	Ovarian cancer	miR‐7	Oncogene	miRNA target erasers 3′UTR	HuR‐dependent (cell)	Stimulate EGFR‐PI3K/AKT/mTOR pathway	72

Abbreviations: MeRIP‐qPCR, methylated RNA immunoprecipitation quantitative polymerase chain reaction; NSCLC, non‐small cell lung cancer; processing↑, promote pri‐miRNAs processing; processing↓, inhibit pri‐miRNAs processing.

Though FTO has always been a high‐profile topic for scientists, literature about FTO and miRNAs remains few. Since FTO's multiple functions, the interrelationship between FTO and miRNAs catalysis are not limited in an m6A‐dependent manner. In HER‐2 positive breast cancer, highly expressed FTO was observed to reduce miR‐181b‐3p and drove migration and invasion effects through miR‐181b‐3p/ADP ribosylation factor‐like 5B(ARL5B) axis. Nonetheless, it is not demonstrated that FTO regulated miR‐181b‐3p level in an m6A‐dependent way since no observation of FTO‐mediated miR‐181b‐3p m6A alteration based on MeRIP‐seq result.^69^ It was also found that FTO mRNA was identified as a downstream target of miR‐607 in LUAD. miR‐607 was suppressed by Circ‐0072309 through miRNA response elements (MREs), which restored FTO expression and consequently facilitated LUAD tumorigenesis and invasion.^70^

Research concerning another demethyltransferase ALKBH5 faced poor production as well. Available literature elucidated that ALKBH5 was involved in cancer cell growth and apoptosis in an m6A‐dependent way. In osteosarcoma, ALKBH5 was revealed to remove m6A from pre‐miR‐181b‐3p. Therefore, YTHDF2, a degradation‐facilitating m6A reader, failed to degrade pre‐miR‐181b‐3p without m6A modifications. Eventually, this combination of m6A regulators upregulated miR‐181–3p to repress YAP, promoting apoptosis and attenuating malignancy phenotype.^71^ In an addition, if ignoring the YTHDF2's degradation impact, this article provided a novel sight of ALKBH5's promoting effect on miRNA maturation while decreasing m6A levels. On the other hand, it is implied that ALKBH5 cooperated with miR‐193a‐3p to aggravate oesophageal carcinoma (ESCC) progression. Notably, further evidence indicated that miR‐193a‐3p could silence ALKBH5, and simultaneously ALKBH5 impeded miR‐193a‐3p processing in an m6A‐dependent way in turn. In another word, ALKBH5 and miR‐193a created a positive feedback loop, verifying the existence of m6A‐miRNAs mutual regulation and intensifying oncogenic effects.^47^ The same combination but in glioma, while ALKBH5 was only screened as a target of miR‐193a‐3p. ALKBH5 positively regulated AKT2, a key molecule that inhibits the intrinsic apoptosis signalling pathway. As miR‐193a‐3p alleviated, restored‐ALKBH5 facilitated the AKT2 pathway to exert an apoptosis‐promoting effect.^49^ In epithelial ovarian carcinoma, it is elucidated that ALKBH5 suppressed miR‐7 processing and drove a tumour‐promoting effect. However, in this study, ALKBH5 reduced miR‐7 expression in a HuR‐relative way, instead of the m6A‐dependent one.^72^

Resembling methyltransferases, demethyltransferases also drive dual effects in diverse cancers, especially when they link to multiple readers and downstream pathways. Surprisingly, ALKBH5 exhibited both effects of promoting miRNAs processing and degradation. Researchers provide a reasonable explanation that ALKBH5 accelerated miRNAs processing when cooperating with decay‐inducing reader YTHDF2. Hence, in terms of m6A‐associated miRNA metabolism, it could not be ignored that m6A readers are the ones who dominate m6A functions beyond writers and erasers. There is thereby an urgent necessity to comprehensively consider m6A regulators' combined effects on miRNAs processing.

## 6 COMBINED ROLES OF M6A READERS AND MIRNAS IN CANCERS

RNA‐binding proteins possess multiple types and functions, taking charge of recognizing m6A modification and guiding corresponding metabolic processes. Similarly, the phenomenon of readers' dual functions toward mRNAs also exists when it comes to miRNAs, including processing‐promoting and degradation‐promoting. Even though methyltransferase and demethyltransferase are in charge of altering m6A levels, readers are the decisive factors that devote to real functional implementation of m6A modification. It is obvious that m6A readers' dual functions and writer/eraser m6A regulatory effects complicate the conditions of miRNAs biosynthesis and cancer‐related physical behaviours (Table 5).

**TABLE 5:** The interaction between m6A readers and miRNAs

M6A regulators	Cancer type	miRNA	Readers' role	Mechanism	Validation approach	Function of readers	Ref
HNRPRA2B1	‐	‐	‐	Processing↑	m6A‐seq and HNRNPA2B1‐ HITS‐CLIP (cell)	‐	41
HNRPRA2B1	NSCLC	miR‐106b‐5p	Oncogene	Processing↑	HNRPRA2B1‐RIP‐qPCR (cell)	Activate CRY2/c‐MYC	73
HNRPRA2B1	Breast cancer	miR‐29a/b, miR‐222/1266/1268a/671‐3p	Oncogene	Processing↑	miRNA‐seq (cell)	Cause endocrine resistance	74
HNRPRA2B1	Oesophageal cancer	miR‐17‐92 cluster	Oncogene	Processing↑	m6A/HNRNPA2B1‐RIP‐qPCR (cell)	Gene ontology (GO) analysis indicate relationship with TGF‐β, p53, Wnt, MAPK and mTOR signalling pathways	89
YTHDF2	Osteosarcoma	miR‐181b‐3p	Suppress	Processing↑	MeRIP‐qPCR (cell)	Inhibit YAP expression	71
YTHDC1	PDAC	miR‐30d	Suppress	Processing↑	m6A/YTHDC1‐RIP‐qPCR (cell)	Stimulate Warburg effect/P/M/angiogenesis and inhibit RUNX, SLC2A and HK1 expression	75
YTHDF2	Ovarian cancer	miR‐145	Oncogene	miRNA targets reader 3′UTR	Dual luciferase report assay, miRNA mimic/inhibitor, clinical data correlation (human, cell)	Global m6A alteration	76
YTHDF2	Prostate cancer	miR‐495	Oncogene	miRNA target reader 3′UTR	Dual luciferase report assay, miRNA mimic/inhibitor, clinical data correlation (human, cell)	Stimulate MOB3B mediated signalling	78
YTHDF2	HCC	miR‐145	Oncogene	miRNA targets reader 3′UTR	Dual luciferase report assay, miRNA mimic/inhibitor, clinical data correlation (human, cell)	Alter m6A level and promote HCC proliferation	77
YTHDF2	Prostate cancer	miR‐493‐3p	Oncogene	miRNA targets reader 3′UTR	Dual luciferase report assay, miRNA mimic/inhibitor, clinical data correlation (human, cell)	Suppress m6A level and promote cell proliferation	90
IGF2BP2	Thyroid cancer	miR‐204	Oncogene	miRNA targets reader 3′UTR	Dual luciferase report assay, miRNA mimic/inhibitor (cell)	Elevate c‐MYC m6A level and accelerate progression	50
YTHDF1	NSCLC	miR‐376c	Oncogene	miRNA targets reader 3′UTR	Dual luciferase report assay, miRNA mimic/inhibitor (cell)	Stimulate Wnt/β‐catenin pathway	79
YTHDF1	Glioma	miR‐346	Oncogene	miRNA targets reader 3′UTR	Dual luciferase report assay, miRNA mimic/inhibitor (cell)	Promote tumour growth and prognosis value	80
HNRNPC	Oesophageal cancer	miR‐186	Oncogene	miRNA targets reader 3′UTR	Dual luciferase report assay (cell)	Promote proliferation, migration and invasion	88

Abbreviations: HCC, hepatocellular cancer; HITS‐CLIP, high through sequence‐crosslinking immunoprecipatation; NSCLC, non‐small cell lung cancer; PDAC, pancreatic ductal adenocarcinoma; processing↑, promote pri‐miRNAs processing; processing↓, inhibit pri‐miRNAs processing; RIP‐qPCR, RNA immunoprecipitation quantitative polymerase chain reaction.

After reporting miRNAs processing in a METTL3 and m6A dependent way, Alarcon et al continued subsequent research of m6A reader HNRNPA2B1. Alarcon and colleagues firstly confirmed that HNRNPA2B1 recognized m6A of pri‐miRNAs and recruited microprocessor complex to facilitate the processing of primary miRNAs.^41^ m6A was commonly identified in the GGAC motif which is usually located near a junction of flanking and stem region of primary miRNAs. This excellent site allows localization of microprocessor complex followed by flanking region slicing. Since HNRNPA2B1's general processing‐promoting effect on miRNAs and miRNAs' universal tumour‐suppressing silencing function, HNRNPA2B1 seemed to act as an oncogenic factor in various cancers. To date, it is uncovered that HNRPRA2B1 took part in cancer cell proliferation and drug resistance aspects. In NSCLC, HNRNPA2B1, cooperating with lncRNA LINC01234, promoted miR‐106b‐5p maturation and drove an aggressive effect. Cryptochrome 2(CRY2) was identified to be a tumour suppressor and upstream inhibitor of oncogenic c‐MYC. Enhanced miR‐106b‐5p attacked CRY2 and hence stabilized c‐MYC, accelerating cell proliferation and resulting in poor clinical outcomes.^73^ In addition, breast cancer tamoxifen‐resistant LCC9 cells exhibited highly expressed HNRNPA2B1. To investigate whether HNRNPA2B1 induced endocrine resistance in breast cancer cells, researchers constructed HNRNPA2B1‐overexpressed MCF‐7 cells (formerly tamoxifen sensitive). It is confirmed that excessive HNRNPA2B1 eliminated the ability of endocrine blockers like tamoxifen, supporting its important role in endocrine‐resistance breast cancer. Subsequently, global miRome analysis and qPCR had been performed to confirm the downregulation of selected miR‐29a‐3p, miR‐29b‐3p and miR‐222, and upregulation of selected miR‐1266‐5p, miR‐1268a and miR‐671–3p in the HNRNPA2B1‐overexpressed MCF‐7 cell.^74^ It is somewhat astonishing that HNRNPA2B1 was also capable to downregulate miRNAs. It is possible to hypothesize that this condition is likely to occur when there were more intermediate links or different patterns involved.

Comparable to HNRNPA2B1, YTHDC1 and NKAP could decode m6A and exhibit pri‐miRNAs processing effect as well. In consideration that NKAP has been illustrated in the former part,^44^ here is no more detailed description. YTHDC1 was also suggested to expedite miR‐30d processing in PDAC and was involved in cancer‐associated metabolism reprogramming Warburg effect. Mechanically, miR‐30d bound to transcriptional factor RUNX, which failed to evoke glucose transporter‐encoding SLC2A and hexokinase (HK1) transcription. Therefore, YTHDC1 attenuated PADC aerobic glycolysis, repressing PDAC occurrence and progression through pri‐miR‐30d processing.^75^

As mentioned before, another known m6A reader YTHDF2 could destabilize m6A‐containing RNAs, exerting a negative effect on RNA stability and subsequent procedure. As for miRNAs, YTHDF2 impeded their processing through degradation as well. According to the aforementioned research, YTHDF2 failed to decay pre‐miR‐181 when ALKBH5 removed m6A, while YTHDF2 degrade pre‐miR‐181 when it received m6A again.^71^ In addition to that YTHDF2 could degrade miRNAs, YTHDF2 was also confirmed to be the target of diverse miRNAs. In ovarian cancer and HCC, YTHDF2 was both screened as a miR‐145 target object and functioned as an oncogenic factor. Since miR‐145 was repressed, enhanced‐YTHDF2 destabilized m6A‐carrying RNAs, contributing to cancer cell proliferation, apoptosis inhibition and migration.^76^, ^77^ In prostate cancer, YTHDF2 was also suppressed by miR‐495 and exerted oncogenic influence as well. As reduced miR‐495 failed to silence YTHDF2, elevated‐YTHDF2 degraded MOB family kinase activator 3B (MOB3B), expediting cell proliferation and distant metastasis. These results consistently suggest that YTHDF2 seemed to tend to drive a tumour‐promoting effect.^78^

From existing literature, no evidence suggests that YTHDF1 and IGF2BP2 exhibited function regarding miRNA slicing processing. On the contrary, anti‐tumour miR‐376c targeted oncogenic YTHDF1 in NSCLC. As miR‐376c silenced YTHDF1, it disrupted the Wnt/β‐catenin pathway, inhibiting NSCLC cell aggressive phenotype and poor progression.^79^ In glioma, YTHDF1 was identified as a miR‐346 downstream target as well. As miR‐346 decreased, accumulating YTHDF1 promoted tumour growth and indicated a poor prognosis.^80^ Alternatively, IGF2BP2 was revealed to be targeted by miR‐204 in thyroid cancer. However, LNR MALAT could sponge with miR‐204, impeding miR‐204 to attack IGF2BP2. Restored‐IGF2BP2 promoted m6A‐c‐MYC translation efficiency, facilitating c‐MYC‐mediated migration, invasion and apoptosis suppression.^50^ Though there is no available source concerning YTHDF1 and IGF2BP2's pri‐miRNAs processing‐promoting function, they contributed to cancer development via miRNAs‐m6A way.

To date, it is found that three readers, including HNRNPA2B1, NKAP and YTHDC1, exhibited miRNAs processing function. There is only YTHDF2 was revealed as a negative reader of miRNAs processing. As for readers' roles in cancers, it is demonstrated that m6A readers and miRNAs' multiple relationships contribute to the occurrence and development of various cancers, relatively showing an oncogenic tendency. Being convinced of readers' dual effects on miRNA processing, it is understandable that the writer could drive both influences on miRNAs like NSun2 and METTL3. On the other hand, demethyltransferase ALKBH5 is somehow able to exhibit both processing‐promoting and degradation effects. However, for now, a majority of current reports separated m6A readers from research content, and depicted writers or erasers unilaterally. Exploring their individual superficial devotions narrows our horizon against the detailed miRNAs biosynthesis regulatory mechanism in an m6A‐dependent way. These results had an important implication for the developing conceptual premise that the m6A modification function concerning pri‐miRNAs processing depends on m6A levels alteration and readers' decisive recognizing effects. The exertion of the m6A modification function lies in the relative expression of these three types of regulators, commanding a rigorous and scientific attitude toward experiment and analysis.

## 7 THE EXPECTATION FOR THERAPEUTIC VALUE OF M6A AND MIRNAS

As we investigate closely on literature concerning m6A and miRNAs, we find out that more attention has been focused on clinical personality treatment. A comprehensive analysis of the combined effects of m6A regulators and miRNAs supports that certain combination indeed facilitates tumour malignancy or suppress tumour progression. Molecule‐specific mediated therapy is one of the foundations of precision medicine. In this case, it is suggested that m6A has inestimable clinical value via utilizing blocking agents to provide precise attacks and applying analogues for specific deficiency compensation (Table 6). In gallbladder cancer, DAC exhibited a possible therapeutic value for those with METTL3 high expression through disturbing METTL3/miR‐92b‐3p related cancer progression.^54^ In breast cancer, metformin exerted an anti‐growth effect via reinforcing miR‐483‐3p level, which could silence oncogenic METTL3 and restore apoptosis‐related p21. Consequently, metformin might be clinically potential to apply to METTL3‐overexpressed breast cancer patients.^48^

**TABLE 6:** M6A‐miRNAs based targeting therapy

miRNA	Cancer type	M6A regulators	Mechanism	Ref
miR‐92b‐3p	Gallbladder cancer	METTL3	DAC inhibit METTL3/miR‐92b‐3p related cancer progression	54
miR‐483‐3p	Breast cancer	METTL3	Metformin exerts anti‐growth effect via METTL3/miR‐483‐3p	48
miR‐17‐92 cluster	Gastric cancer	METTL3	Overexpressed METTL3 accelerates everolimus sensitivity by stimulating mTOR pathway	81
miR‐29a/b, miR‐222/1266/1268a/671‐3p	Breast cancer	HNPNPA2B1	HNPNPA2B1 inhibitor might alleviate endocrine resistance	74
miR‐221‐3p	Breast cancer	METTL3	Excessive METTL3 drive Adriamycin resistance	82

What is more, according to available research findings, aberrant m6A regulators and miRNAs amount may imply certain drug sensitivity or drug resistance phenomena. It is well‐known that drug resistance is the main challenge of anti‐tumour treatment all the way long. Under this circumstance, m6A regulators could enhance therapeutic response and alleviate drug resistance, laying a foundation for combination therapy. m6A‐dependent miR‐17‐92 cluster biogenesis activated the mTOR pathway, accelerating mTOR inhibitor everolimus sensitivity in gastric cancer.^81^ mTOR inhibitor everolimus is now applied in advanced renal cancer patients. If the METTL3/AKT/mTOR signalling exists in renal cancer as well, the patients with overexpressed METTL3 might benefit more from everolimus. Carolyn M. Klinge and colleagues reported that overexpressed HNPNPA2B1 is involved in endocrine resistance, which suggested that HNPNPA2B1 inhibitors might be applied to alleviate endocrine resistance to improve clinical efficacy.^74^ In breast cancer, excessive METTL3 resisted cell apoptosis as well as exhibited Adriamycin resistance. METTL3‐induced mature miR‐221‐3p bound to HIPK2 3′UTR, attenuating to repress drug resistance‐related Che1 expression and finally driving Adriamycin resistance. METTL3 inhibitor might provide a reference for treatment.^82^

Serious research has provided evidence about targeted‐based therapy at a cellular level or in animal models. However, a majority problem of m6A‐miRNAs‐based therapy with these studies is how to construct an exchanging platform of basic research and clinical applications. As for miRNA therapeutics, firstly, multiple miRNA targets indicated a major uncertain miRNA‐based therapeutic effect. A contradiction might occur while a particular miRNA targets both oncogenes and tumour suppressors. In this situation, apart from attacking expected targets, the off‐target effect might cause toxicity in other normal tissue or organs.^83^ Furthermore, miRNA delivery vehicle construction is another difficulty in targeting function implementation. An excellent delivery system helps to avoid miRNAs degradation in serum and enables tissue‐specific delivery to alleviate off‐target toxicity.^84^ Finally, the modulatory degree of miRNAs is somehow within a small scope of downstream gene expression, the miRNA‐based therapy effect might not be promising. So, it is a key point to explore how to magnify miRNA‐based drug efficiency before clinical application. And with regard to m6A regulator analogue or antagonist, its clinical research still lags far behind basic research. Similarly, with wide‐ranging implications and dual effects on cancers, m6A‐miRNA‐related targeting therapy still faces the problem of off‐target toxicity. As mentioned above, there is a long way to go before m6A‐miRNAs‐based targeting treatment is available in clinical application.

## 8 CONCLUSIONS

N6‐methyladenosine modification is one of the most prevalent post‐transcriptional epigenetics in mammalian RNAs. This review shares the mutual interactions between m6A regulators and miRNAs; focuses on the combined effect of various m6A regulators and miRNAs in different cancers. m6A regulators could regulate miRNAs processing, meanwhile, miRNAs could attack m6A regulator mRNAs to alter the m6A level in return. Particularly, miRNAs are capable of assisting m6A regulators to better exert their functions through binding to the m6A motif. Notably, m6A readers possess the fateful effects of miRNAs biosynthesis, transcending writers and eraser's m6A abundance alteration functions. Future research regard to m6A‐related miRNAs processing should concentrate on the combined effect of three types of m6A regulators.

**FIGURE 3:**
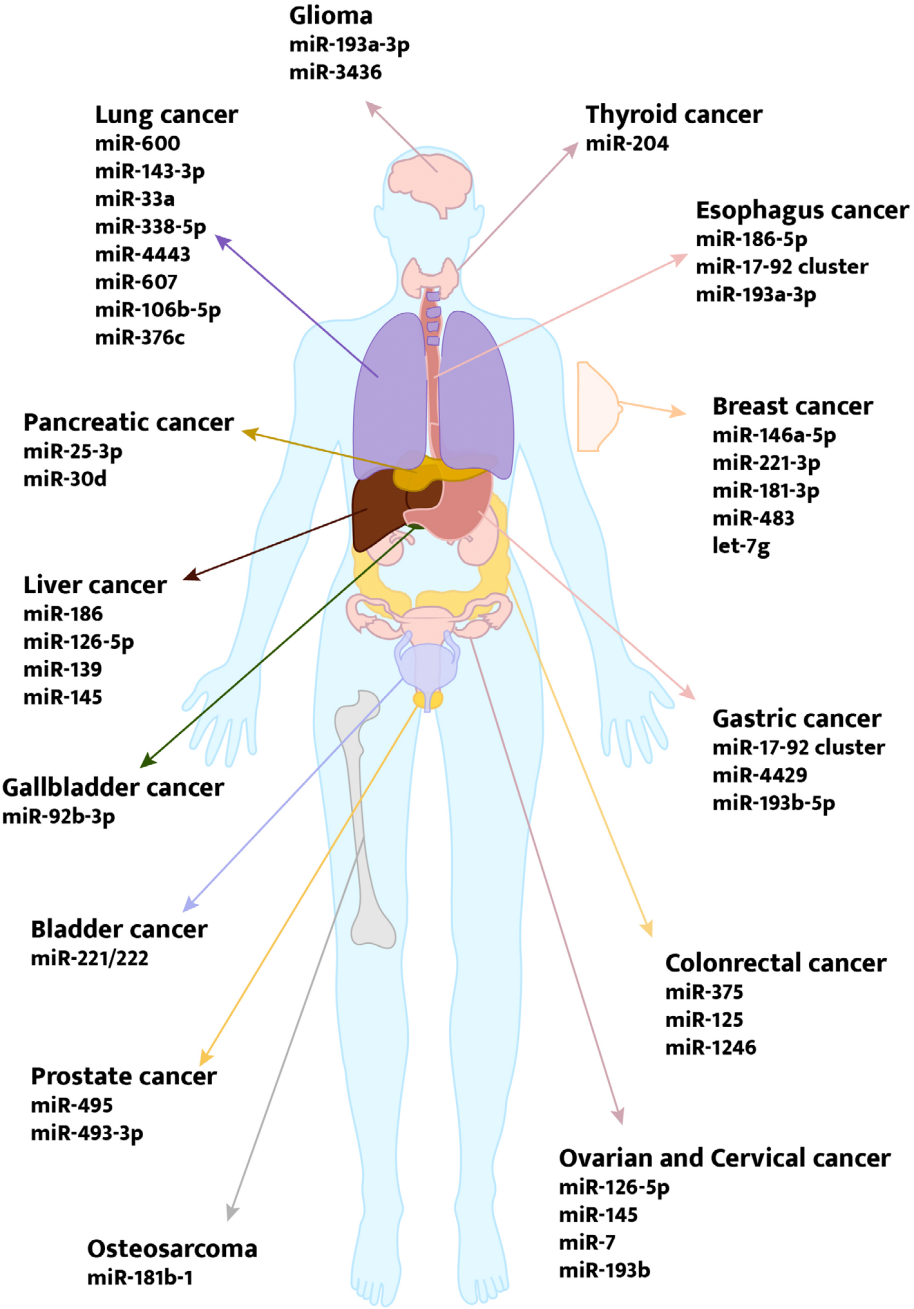
M6A modification and miRNAs are involved in various cancers. Articles concerning m6A and miRNAs cover the digestive system, respiratory system, urogenital system, neural system, skeletal system and endocrine system

From the digestive system, respiratory system, productive system, neural system, skeletal system and endocrine system (Figure 3), m6A and miRNAs contribute to cancer involving aspects converting from self‐sufficient growth signals, resisting cell death, tissue metastasis, and invasion as well as deregulating cell energy. Theoretically, different combinations of m6A regulators and miRNAs and downstream pathways create infinite possibilities. Indeed, the role of m6A modification in miRNA‐relative cancer depends on its function and location within the whole signalling, affected by miRNAs regulative effects as well as subsequent pathways. Though it is reasonable that m6A regulators are like a double‐edged sword in cancers, existing published literature indicated certain trends.

For the reason that aberrant N6‐methyladenosine level contributes to cancer malignancy and clinical deterioration, m6A modification exhibited potential therapeutic value. However, subject to the existing limited acquaintance and complexity of m6A regulators and miRNAs, it is a huge challenge to apply to clinical medicine from available reports. Consequently, further and overall studies are indispensable for paving a way for clinical applications.

## AUTHOR CONTRIBUTIONS

Huiru Feng and Fan Wang contributed to the conception and design of this work. Xiong Liu supervised the study. All authors contributed to the collection and interpretation of literature and writing, review and revision of the manuscript. All authors read and approved the final manuscript.

## FUNDING INFORMATION

This work was supported by the National Natural Science Foundation of China (grant number 81902774); the Natural Science Foundation of Guangdong Province (grant number 2020A1515010176); the Medical Scientific Research Foundation of Guangdong Province of China (grant number A2020078); and the Presidential Foundation of Nanfang Hospital (grant number 2017C00).

## CONFLICT OF INTEREST

The authors declare no competing interests.

## Data Availability

The data that support the findings of this study are available from the corresponding author upon reasonable request.
